# Inter-rater reliability of the evaluation of muscular chains associated with posture alterations in scoliosis

**DOI:** 10.1186/1471-2474-13-80

**Published:** 2012-05-28

**Authors:** Carole Fortin, Debbie Ehrmann Feldman, Clarice Tanaka, Michelle Houde, Hubert Labelle

**Affiliations:** 1 Centre de recherche du CHU Sainte-Justine, Montréal, Québec, Canada; 2 Faculté de médecine, Université de Montréal, Montréal, Québec, Canada; 3 École de réadaptation, Faculté de médecine, Université de Montréal, Montréal, Québec, Canada; 4 Groupe de Recherche Interdisciplinaire en Santé, Montréal, Québec, Canada; 5 Department of Physiotherapy, Communication Science & Disorders and Occupational Therapy, Faculty of Medicine, University of São Paulo, São Paulo, Brazil; 6 Institut de recherche du Centre universitaire de santé McGill, Montréal, Québec, Canada

**Keywords:** Posture, Reliability, Muscular chain

## Abstract

**Background:**

In the Global postural re-education (GPR) evaluation, posture alterations are associated with anterior or posterior muscular chain impairments. Our goal was to assess the reliability of the GPR muscular chain evaluation.

**Methods:**

Design: Inter-rater reliability study. Fifty physical therapists (PTs) and two experts trained in GPR assessed the standing posture from photographs of five youths with idiopathic scoliosis using a posture analysis grid with 23 posture indices (PI). The PTs and experts indicated the muscular chain associated with posture alterations. The PTs were also divided into three groups according to their experience in GPR. Experts’ results (after consensus) were used to verify agreement between PTs and experts for muscular chain and posture assessments. We used Kappa coefficients (K) and the percentage of agreement (%A) to assess inter-rater reliability and intra-class coefficients (ICC) for determining agreement between PTs and experts.

**Results:**

For the muscular chain evaluation, reliability was moderate to substantial for 12 PI for the PTs (%A: 56 to 82; K: 0.42 to 0.76) and perfect for 19 PI for the experts. For posture assessment, reliability was moderate to substantial for 12 PI for the PTs (%A > 60%; K: 0.42 to 0.75) and moderate to perfect for 18 PI for the experts (%A: 80 to 100; K: 0.55 to 1.00). The agreement between PTs and experts was good for most muscular chain evaluations (18 PI; ICC: 0.82 to 0.99) and PI (19 PI; ICC: 0.78 to 1.00).

**Conclusions:**

The GPR muscular chain evaluation has good reliability for most posture indices. GPR evaluation should help guide physical therapists in targeting affected muscles for treatment of abnormal posture patterns.

## Background

Correction of posture is an important aim of physical therapy for persons with orthopaedic or neurologic impairments. Posture alterations can be associated with modifications in muscular moments which can change joint alignment and cause movement impairment syndromes [1]. These impairments can affect functional activities and restrict the active life [2]. Sahrmann [3] states that the evaluation of posture leads to the understanding of the impact of muscle imbalance on the observed posture alterations. Thus, physical therapists must work on reducing these imbalances (releasing muscle tension and tightness and increasing muscle strength) in order to improve posture.

Various authors have described methods for evaluation of muscle action in relation to posture alterations [1,4-9]. Muscle imbalance associated with posture alterations are explained by the anatomy and physiology of the involved muscles [1,5]. Kendall et al. [5] and Sahrmann [1] refer to stiffness and muscle weakness associated with posture alterations. Souchard [6] describes muscles as being organized into two main static postural chains: the anterior and posterior muscular chains. Muscular chains are an ensemble of muscles defined according to their localization as well as their functional role which can explain posture alterations and movement dysfunctions [6,10-12]. Specific posture patterns caused by muscle chain retractions have been associated with lower back or neck pain among elite athletes in muscular power competitions [12] and functional disabilities in an adult with hemiparesis [13]. Despite the lack of studies linking muscular chain impairments to abnormal posture patterns and dysfunction, it seems that global muscular chain stretching is more effective than analytic muscle stretching to improve function and quality of life for several pathologic conditions including respiratory, musculoskeletal and neurological problems [7-9,13-15].

According to Souchard [6], it is important to evaluate posture in a global fashion in order to establish appropriate treatments that target muscles in these muscular chains. This method is referred to as Global Postural Re-education (GPR) [6]. Souchard [6] divides postural evaluation into three components. The first part, called general photography, allows for identification of the person’s morphology type – i.e. anterior, posterior or mixed. For example an anterior type would present with shoulders rolled forward, an exaggeration of sagittal vertebral curves and valgus of the lower limbs. A posterior type would typically present as someone with elevated shoulders, decrease in sagittal vertebral curves and varus of the lower limbs. A mixed type presents anterior and posterior characteristics. The second part of the evaluation, “examination of retractions”, is based on observation of posture in the standing position. The aim of this evaluation is to identify anterior or posterior muscles responsible for the observed posture alterations. The third part of the evaluation involves the possibility for correction of the posture alterations and compares posture in standing and sitting positions. Comparison between standing and sitting posture helps determine which muscular chain (anterior or posterior) contributes more to a specific posture alteration. These different steps allow the clinician to select the necessary stretching postures and sensorimotor integration exercises to be used for treatment. The evaluation is essential as it guides the clinician in his/her comprehension of the effects of muscle action on the observed posture alterations. Documenting impaired posture is also recommended in the Guide to Physical Therapist Practice [16].

Actual posture assessment tools (both in the laboratory and in the clinical setting) allow for quantification of observed posture alterations, producing numerical indices [17-24]. However, they do not provide guidance in interpreting muscle imbalances that are inherent in the various posture alterations. The evaluation of muscles related to posture alterations is helpful to identify and understand the inherent muscular causes as well as for selecting appropriate posture re-education exercises. To our knowledge, the reliability of the GPR muscle component evaluation has not been reported. Thus, the objectives of our study were 1) to determine the inter-rater reliability of the evaluation of muscular chains and their associated posture alterations, from photographs of adolescents presenting with idiopathic scoliosis; 2) to verify the impact of clinical experience on the level of reliability of muscular chain evaluation; and 3) to verify the agreement of posture and muscular chain evaluations observed by physical therapists and two experts instructors in GPR.

## Methods

### Participants

We recruited 50 physical therapists (PTs) trained in GPR from Canada, Europe and Brazil for this study. Two physical therapists instructors in GPR served as experts for determining muscular chain impairments associated with posture alterations, in the absence of any objective “gold standard” criterion for this assessment. Muscular chain impairments were determined by the two experts according to standards taught in GPR which can be found in GPR literature [6,7,13,14]. Each PT used a 23 item posture analysis grid (described below) to assess standing posture of five youths aged between 13 and 20 years old with idiopathic scoliosis (Cobb angle 15 to 40°). Youths with idiopathic scoliosis were chosen because they typically display posture alterations [18,23]. These youths (4 females, one male) were recruited from a previous study on posture assessment performed at the Sainte-Justine University Hospital Center in Montreal. Mean age of youths was 15.6 ± 2.2 years and average weight and height were 53.6 ± 11 Kg and 161.9 ± 13.8 cm, respectively. Two youths had a right thoracic scoliosis (33° and 36°), two had a double major scoliosis (18°-15° and 23°-24°) and one had a left thoraco-lumbar scoliosis (38°). We selected youths with different morphological characteristics (anterior, posterior or mixed) with clear photographs. We used photographs for the posture assessment since they are a rapid and accurate way to assess posture [17,19,21,24-26]. Physical therapists, experts, youths with idiopathic scoliosis and their parents signed informed consent forms and the project was approved by the ethics committee of Sainte-Justine University Hospital Center.

### Description of the posture analysis grid

PTs were asked to determine posture alterations from a posture analysis grid that was based on the Tyson and Desouza content validity study [2] and on reliability of the grid already reported in previous studies [24-26]. This posture analysis grid contains 23 posture indices representing the six body regions (head and neck, shoulders and scapulae, thoracic region, lumbar region, pelvis and lower limbs) (see Table 1 for more details).

**Table 1:** Posture Analysis Grid

**Body segments**	**Observations**	**Muscular chain**			
		**Ant.**	**Post.**	**Ant. and Post.**	**N.E.**
		**(1)**	**(2)**	**(3)**	**(4)**
**Head**	**N □ Protraction □**	□	□	□	□
	**Lateral bending R □ L □**	□	□	□	□
	**Rotation R □ L □**	□	□	□	□
**Cervical**	**Lordosis > □ N □** **< □**	□	□	□	□
**Shoulders**	**N □ Elevated R □L □ R > L □ R** **< L □**	□	□	□	□
	**Protracted R □ L □R > L □ R < L □ Rounded R □L □ R > L □ R** **< L □**	□	□	□	□
		□	□	□	□
**Scapulas**	**N □ Adducted R □ L □R > L □ R < L □**	□	□	□	□
	**Abducted R □ L □R > L □ R < L □**	□	□	□	□
**Thoracic**	**Kyphosis > □ N □ < □**	□	□	□	□
	**Trunk list C7/S1 : To the R □** or **L □**				
	(From back)				
**Lumbar**	**Lordosis > □ N □ < □**	□	□	□	□
**Pelvic**	**N □ Frontal tilt R □ = □L □**	□	□	□	□
	**Anteversion R □ L □R > L □ R < L □**	□	□	□	□
	**Retroversion R □ L □R > L □ R < L □**	□	□	□	□
**Lower limbs**					
**Knee**	**N □ Valgus R □ L □R > L □ R < L □**	□	□	□	□
	**□Recurvatum R □ L □R > L □ R < L**	□	□	□	□
	**□ Flexum R □ L □R > L □ R < L □**	□	□	□	□
**Feet**	**N □ Arch ↙ R □L □ R > L □ R** **< L □**	□	□	□	□
	**Arch ↗ R □ L □R > L □ R < L □**	□	□	□	□
	**Valgus R □ L □R > L □ R < L □**	□	□	□	□
	**Varus R □ L □R > L □ R < L □**	□	□	□	□

Legend : N = normal; R = right; L = left; Arch **↙**= decreased plantar arch; Arch **↗** = increased plantar arch; Ant. = anterior; Post. = posterior; N.E. = no evaluable.

### Procedure

All evaluations were done via a web site. Each PT had access to consent forms, photographs of the five youths, posture analysis grid and explanations about the procedure. There were seven photographs per person representing different standing views: anterior, posterior, lateral right and left (with and without arms flexed) and one was taken in an oblique position (45°) to help with visualisation of the sagittal vertebral curvatures. Each photograph could be focussed to better see a specific body segment. The first step was to note the presence or absence of posture alteration for each of the 23 posture indices. When the posture alteration was present on both sides (for example protracted shoulder), the PT had to indicate if the alteration was equal or greater on one side. Secondly, the PT had to determine if the posture alteration was attributable to retraction of muscles in the anterior muscular chain (1), posterior muscular chain (2), anterior and posterior muscular chains (3) or unable to be evaluated (4) (see Table 1 for more details). The two experts completed the same procedure. If there was disagreement between the two experts, they discussed their results to reach a consensus. If a consensus could not be reached, a third expert (CF) made the final decision. After consensus, agreement between PTs and experts was calculated.

### Data analysis

We used Fleiss’ Kappa coefficients (for categorical data) and percentage of agreement to assess inter-rater reliability of muscular chain evaluation and associated posture alterations (objective 1). Among those who identified the same posture alteration, we determined whether there was agreement on the muscular chain evaluation. For example, among those who recorded knee valgus as an alteration, we assessed the inter-rater reliability of the muscular chain assignment associated with knee valgus. To address our second objective, we divided the PTs into three groups according to their experience in GPR (Group 1: ≤ 2 years, Group 2: 2.5 to 9.5 years and Group 3: ≥ 10 years) and the analysis of the muscular chain associated with posture alterations was made for each of the three groups. We determined whether there were differences between the 3 groups for Kappa coefficients ≥ 0.40, using the chi-squared test (*χ*^2^). The percentage of agreement provides a measure of agreement but unlike the kappa coefficient it doesn’t take into account the agreement obtained by chance [27,28]. Fleiss’s Kappa is used to measure the overall agreement between several raters and is adapted for nominal scales with multiple categories [28-30]. Interpretation of the Kappa coefficients is as follows: values 0.81 – 1.00 = almost perfect; 0.61 – 0.80 = substantial; 0.41 – 0.60 = moderate; 0.21 – 0.40 = fair; 0.01 – 0.20 = slight; ≤ 0 = poor agreement [28,31].

For our third objective, we examined agreement betweens PTs and experts regarding muscular chain evaluation and their associated posture alterations, using intra-class correlation coefficients (ICC_3,k_) for categorical data (agree or disagree). PTs’ answers were re-coded as agree versus disagree with experts’ answers (after consensus) and were averaged for each possible choice of posture indices of the grid (example: right knee flexum – R, left knee flexum – L, R > L or R < L; see Table 1). Interpretations of ICCs were based on Portney and Watkins [32] criteria: > 0.75 = good level of agreement, 0.50 to 0.75 = moderate and < 0.50 = poor level of agreement. We used the Online Kappa Calculator (justusrandolph.net/kappa/) program for the Kappa statistics and SPSS 12.02 for the ICC computation.

## Results

### Inter-rater reliability

For the muscular chain evaluation, the percentage of agreement ranged from 28 to 82% and the level of reliability is moderate to substantial for 12 posture indices (K: 0.42 to 0.76) for the PTs (middle column, Table 2). The percentage of agreement ranged from 67 to 100% and the level of reliability was fair to moderate for three items and perfect for 19 posture indices for the experts before consensus (middle column, Table 2). When PTs are divided into three groups according to their experience in GPR, the level of reliability for muscular chain evaluation is moderate to substantial for 8 posture indices in Group 1 (≤2 y., K: 0.43 to 0.91), for 8 posture indices in Group 2 (2.5 to 9.5 y., K: 0.40 to 0.73) and for 16 posture indices in Group 3 (≥ 10 y., K: 0.41 to 1.00) (Table 3). Group 3 has a significantly higher level of reliability than the other groups (*χ*^2^, p = 0.005). The inter-rater reliability for muscular chain evaluation is higher in the three groups for the following posture indices: head protraction, decreased cervical lordosis, rounded shoulders, decreased kyphosis and pelvis posterior tilt.

**Table 2:** Inter-rater reliability and agreement between PTs and experts of the muscular chain evaluation

**Body regions**	**Reliability**		**Agreement**	**Muscular Chain**
**Posture indices**	**PTs**	Experts*	**PTs vs experts**	
	**Kappa (%A)**	**Kappa (%A)**	**ICC (95% CI)**	
Head : Protraction	0.49 (62)	1.00 (100)	0.55 (−0.27 – 0.95)	Ant
Lateral bending	0.04 (28)	1.00 (100)	0.62 (−0.08 – 0.95)	**
Rotation	0.05 (29)	1.00 (100)	0.82 (0.50 – 0.98)	**
Cervical : > Lordosis	0.11 (33)	1.00 (100)	0.86 (0.60 – 0.98)	Ant and Post
< Lordosis	0.55 (67)	1.00 (100)		Ant
Shoulders : Elevated	0.25 (44)	1.00 (100)	0.83 (0.50 – 0.98)	No answer
Protracted	0.17 (38)	0.50 (75)	0.88 (0.66 – 0.99)	Ant and Post
Rounded	0.65 (74)	1.00 (100)	0.50 (−0.42 – 0.94)	Ant
Scapulas : Adducted	0.43 (57)	1.00 (100)	0.96 (0.88 – 1.00)	No answer
Abducted	0.42 (56)	1.00 (100)	0.84 (0.53 – 0.98)	Ant
Thoracic : > Kyphosis	0.73 (80)	1.00 (100)	0.96 (0.87 – 1.00)	Ant
< Kyphosis	0.76 (82)	1.00 (100)		Post
Lumbar : > Lordosis	0.57 (67)	0.55 (80)	0.89 (0.67 – 0.99)	Ant and Post
< Lordosis	0.22 (41)	1.00 (100)	0.93 (0.79 – 0.99)	Post
Pelvis : Anterior tilt	0.40 (55)	1.00 (100)	0.82 (0.48 – 0.98)	Ant
Posterior tilt	0.60 (70)	1.00 (100)		Post
Knees : Valgus	0.36 (53)	1.00 (100)	0.92 (0.78 – 0.99)	Ant
Varus	0.54 (65)	1.00 (100)	0.92 (0.78 – 0.99)	Post
Recurvatum	0.26 (45)	1.00 (100)	0.98 (0.93 – 1.00)	Ant and Post
Flexum	0.08 (32)	0.40 (67)	0.99 (0.97 – 1.00)	**
Feet : < Plantar arch	0.34 (51)	1.00 (100)	0.94 (0.83 – 0.99)	Ant
> Plantar arch	0.46 (59)	1.00 (100)	0.99 (0.98 – 1.00)	Post
Valgus	0.36 (52)	1.00 (100)	0.91 (0.76 – 0.99)	Ant
Varus	0.60 (69)	1.00 (100)	0.97 (0.92 – 1.00)	Post

Legend: PTs = physical therapists, %A = percentage of agreement, Ant = anterior, Post = posterior, Ant and Post = anterior and posterior; 95% CI = 95% confidence interval. * = Results for the experts before the consensus. ** = Specific for each participant.

**Table 3:** Inter-rater reliability of the muscular chain evaluation for each group of physical therapists according to their experience in Global Postural Re-education

**Body regions**	**Reliability**		
**Posture indices**	**Kappa (%A)**		
	**Group 1**	**Group 2**	**Group 3**
	**(≤ 2 years)**	**(2.5 to 9.5 years)**	**(≥ 10 years)**
	**(n = 21)**	**(n = 16)**	**(n = 15)**
Head : Protraction	0.51 (63)	0.40 (60)	0.56 (67)
Lateral bending	0.15 (37)	0.08 (31)	0.04 (29)
Rotation	0.25 (44)	−0.01 (31)	−0.02 (23)
Cervical : > Lordosis	0.32 (49)	−0.12 (25)	0.05 (29)
< Lordosis	0.51 (63)	0.43 (60)	0.80 (85)
Shoulders : Elevated	0.30 (48)	0.10 (33)	0.23 (43)
Protracted	0.29 (47)	0.11 (33)	0.14 (35)
Rounded	0.61 (71)	0.49 (62)	0.83 (88)
Scapulas : Adducted	0.28 (46)	0.26 (45)	0.86 (89)
Abducted	0.31 (49)	0.39 (54)	0.59 (70)
Thoracic : > Kyphosis	0.91 (93)	0.25 (44)	1.00 (100)
< Kyphosis	0.86 (90)	0.57 (68)	0.81 (85)
Lumbar : > Lordosis	0.79 (84)	0.28 (46)	0.63 (73)
< Lordosis	0.15 (36)	−0.05 (19)	0.69 (76)
Pelvis : Anterior tilt	0.39 (55)	0.24 (50)	0.68 (76)
Posterior tilt	0.50 (63)	0.54 (69)	0.79 (85)
Knees : Valgus	0.34 (50)	0.45 (71)	0.47 (60)
Varus	0.35 (50)	0.59 (69)	0.79 (85)
Recurvatum	0.21 (41)	0.23 (36)	0.18 (38)
Flexum	0.26 (27)	0.09 (41)	0.01 (26)
Feet : < Plantar arch	0.30 (48)	0.26 (45)	0.41 (55)
> Plantar arch	0.29 (47)	0.29 (46)	0.66 (74)
Valgus	0.43 (57)	0.26 (45)	0.34 (50)
Varus	0.39 (55)	0.73 (80)	0.76 (82)

The percentage of agreement and Kappa coefficients (K) for the visual observation of posture from photographs are provided in the middle column of Table 4 for PTs and for the two experts before consensus. The percentage of agreement is ≥ 50% for 17 out of 23 posture indices and the level of inter-rater reliability is moderate to substantial for 12 out of 23 posture indices (K: 0.42 to 0.75) for the group of PTs. Except for knee flexum, the percentage of agreement is ≥ 60% for all posture indices and the level of reliability is moderate to perfect for 18 out of 23 posture indices for the two experts. The inter-rater reliability is higher for head protraction, pelvis posterior tilt, knee varus and for foot increased plantar arch and foot varus.

**Table 4:** Inter-rater reliability and agreement between PTs and experts of visual observations of posture from photographs

**Body regions**	**Reliability**		**Agreement**
**Posture indices**	**PTs**	Experts*	**PTs vs experts**
	**Kappa (%A)**	**Kappa (%A)**	**ICC (95% CI)**
Head : Protraction	0.75 (88)	1.00 (100)	0.71 (0.17 – 0.97)
Lateral bending	0.42 (61)	0.55 (80)	0.97 (0.93 – 1.00)
Rotation	0.18 (45)	0.55 (80)	0.88 (0.67 – 0.99)
Cervical Lordosis	0.29 (53)	1.00 (100)	0.78 (0.38 – 0.97)
Shoulders : Elevated	0.30 (48)	1.00 (100)	0.85 (0.58 – 0.98)
Protracted	0.13 (35)	0.00 (60)	0.81 (0.46 – 0.98)
Rounded	0.03 (27)	0.55 (80)	0.11 (−2.15 – 0.87)
Scapulas : Adducted	0.44 (58)	0.55 (80)	0.96 (0.89 – 1.00)
Abducted	0.34 (50)	0.00 (60)	0.90 (0.70 – 0.99)
Thoracic Kyphosis	0.39 (50)	0.55 (80)	0.95 (0.85 – 0.99)
Trunk list	0.59 (71)	0.55 (80)	0.26 (−1.10 – 0.91)
Lumbar Lordosis	0.45 (63)	1.00 (100)	0.95 (0.85 – 0.99)
Pelvis : Frontal pelvic tilt	0.20 (47)	0.00 (60)	0.60 (−0.15 – 0.95)
Anterior tilt	0.25 (44)	1.00 (100)	0.97 (0.92 – 1.00)
Posterior tilt	0.61 (71)	1.00 (100)	0.97 (0.92 – 1.00)
Knees : Valgus	0.48 (61)	1.00 (100)	0.96 (0.88 – 1.00)
Varus	0.65 (74)	0.55 (80)	0.99 (0.98 – 1.00)
Recurvatum	0.55 (66)	0.00 (60)	0.94 (0.82 – 0.99)
Flexum	0.47 (60)	-0.15 (40)	0.98 (0.95 – 1.00)
Feet : < Plantar arch	0.33 (50)	0.55 (80)	0.97 (0.90 – 1.00)
> Plantar arch	0.72 (79)	1.00 (100)	1.00 (0.99 – 1.00)
Valgus	0.38 (53)	0.55 (80)	0.98 (0.94 – 1.00)
Varus	0.63 (72)	1.00 (100)	0.99 (0.96 – 1.00)

Legend: PTs = physical therapists, %A = percentage of agreement, 95% CI = 95% confidence interval, * Results for the experts before the consensus.

### Agreement with experts

Agreement of muscular chain assessment associated with posture alterations is good for 18 out of 21 posture indices (ICCs ranged from 0.82 to 0.99, see Table 2, third column). The level of agreement is moderate for head protraction, head lateral bending and rounded shoulders (ICCs: 0.55, 0.62 and 0.50 respectively). Agreement between PTs and experts for visual observation of posture from photographs is also good for 19 out of 23 posture indices (ICCs from 0.78 to 1.00, see Table 4, third column). There is a moderate level of agreement for head protraction and frontal pelvic tilt (ICCs = 0.71 and 0.60, respectively). The agreement is poor for rounded shoulders (ICC = −0.11) and trunk list (ICC = 0.26).

### Muscular chain impairments associated with posture alterations

The muscular chain could be determined for the majority of posture alterations (see Table 2, fourth column). However, PTs and the two experts did not attribute specific muscular chain impairment for head lateral bending, head rotation and knee flexion. No muscular chain impairment has been identified for elevated shoulder and adducted scapulae since no such alterations were reported in the five cases that were evaluated and the term “no answer” is thus written in the table under these posture indices.

## Discussion

The goal of posture assessment in GPR is to determine muscles that are responsible for the posture alterations and to plan treatment consisting of stretching postures (for anterior or posterior muscles) to increase muscle flexibility and sensorimotor integration exercises to correct posture. In our study, we investigated inter-rater reliability of the muscular chain evaluation associated with posture alterations among physical therapists and assessed agreement with experts in GPR.

We found a moderate to substantial level of reliability for 12 out of 23 posture indices and a good level of agreement with the two experts in GPR for 18 out of 21 posture indices for the muscular chain assessment. The muscular chain impairment associated with posture could be determined for the majority of indices. However, there was a low level of reliability among the PTs for muscular chain assessment associated with head lateral bending, head rotation, increased cervical lordosis, protracted shoulder and knee flexum. In line with these findings, muscular chain impairment associated with posture alterations was not determined for head lateral bending, head rotation and knee flexum. These results are corroborated by the low level of reliability of these indices in our study and in previous reliability studies on visual observation of posture [33-35]. Except for knee flexum index, our experts (before consensus) had similar percentage of agreement results as those reported by Watson and MacDonncha [26] for qualitative observation of posture indices. To our knowledge, no previous study has reported the psychometric properties of muscular chains evaluation associated with posture alterations. Moreover, no true “gold standard” criterion could be used to assess the validity of this concept. However, the good level of agreement between the PTs and experts may reflect the uniform standards taught in GPR.

The poor levels of inter-rater reliability may be attributable to the importance of the 3D component needed to assess these posture indices which is not really possible from photographs (even when using different views like in this study). The magnitude of the posture alterations may be another factor. It is possible that some posture alterations were too discrete to be visually identified. For example, head lateral bending is always associated with some degrees of flexion or extension and rotation [36]. It might be confusing to determine which component (lateral bending, flexion, extension or rotation) is present when alteration of head position is small and therefore difficult to select the responsible muscular chain. Head lateral bending and rotation can be attributed to anterior muscles such as scalenius, SCM or posterior muscles such as upper trapezius, levator scapulae and cervical erector spinae [1,5,9,37]. PTs trained in GPR can compare posture alterations in the standing and “long sitting” positions to determine whether anterior or posterior muscles are implicated in the posture alteration [7,12,13,38].

For knee flexum, the two experts could not reach a consensus and a third expert had to make the final decision. One expert drew a line to assess this posture index while the other estimated it visually. Differences in their methods combined with discrete posture alterations among the youths for this index may explain the discrepancy between the experts as well as the low and negative kappa coefficients reported respectively for muscular chain and posture evaluation. It may also explain the difficulty to determine inherent muscular chain impairment.

The lack of a clear definition of protracted and rounded shoulder may also explain poor inter-rater reliability among the PTs and the experts. This inconsistency is confirmed by the negative level of agreement between PTs and experts found in our study for rounded shoulder evaluation [39]. In both posture alterations, the shoulder (acromion) can appear forward but muscle implication is different [1]. The protracted shoulder is associated with shorter pectoralis minor muscle whereas rounded shoulder is caused by retraction of pectoralis major muscle and/or serratus anterior muscle [1,4]. These two different concepts are often used in an interchangeable way or are not well defined in the literature [1,5,37,40-42].

We found that more experienced physical therapists (Group 3) had better level of reliability for muscular chain evaluation. This is in contrast with previous studies on visual observation of posture or of gait analysis who reported that inexperienced raters achieve a comparable level of reliability than more experienced raters [43,44]. The PTs in Group 1 and Group 2 had similar results. Group 2 was the most heterogeneous group in terms of nationality: it is possible that more discrepancies exist between physical therapists trained in different countries.

### Study limitations

We used photographs to assess posture. As already mentioned, photographs are a 2D perspective of a person and PTs are used to doing bony palpation when they want to validate their visual observations. However, our main goal was to assess muscular chain impairment associated with the posture alterations. Thus agreement for muscular evaluation was verified for PTs that identified the same specific posture alteration. Another limitation concerns the number of categories for each posture index and for muscular chain evaluation in our assessment scale. Increasing the number of categories in a measurement scale decreases the Kappa coefficients [28]. The analysis for the posture indices was done by combining two to four elements for each of the 23 indices. For example, in the case of knee flexum index, the PT had four choices on the grid (right knee flexum – R, left knee flexum – L, R > L or R < L; see Table 1). In order to have perfect agreement, all the physical therapists would have had to choose all of the same choices (2 to 4 choices) for each of the indices. When we compare the grid for the two experts, there are only a few differences. For the 79 items (representing the 23 posture indices) the two experts are in perfect agreement for 59 of the 79 items; there is a difference for one youth on 17 items and on 3 items for two youths. Thus we have been much more conservative by choosing to analyze the grid by 23 posture indices instead of 79 individual items, and our coefficients are considerably lower as a result. Moreover, Kappa coefficients are less favourably influenced by a large number of raters than are ICCs. The Kappa coefficient is a conservative measure since it eliminates agreement by chance. This explains a Kappa coefficient of zero even if the two experts agreed for three youths out of five and had a percentage of agreement of 60% (Table 4). The small sample of youths included in this study is also a limitation since Kappa coefficients are more favourably influenced by sample size magnitude than by large number of raters [28]. Moreover, some posture alterations were not present among these youths and thus muscular chain impairment could not be determined.

### Clinical applications and recommendations

We found that the muscular chain evaluation (done by PTs trained in GPR) is reliable for most posture indices among youths with idiopathic scoliosis and for the most part, there was good agreement with experts in GPR. This kind of assessment may have considerable diagnostic and therapeutic utility in physical therapy practice as it guides the understanding of muscular impairment associated with abnormal posture patterns. It may assist the physical therapist in the selection of anterior and/or posterior muscular chain stretching exercises to improve posture and increase quality of life [7-9,13,14].

Some adjustments in the teaching of muscular chain evaluation are necessary to improve agreement between physical therapists for less reliable posture indices. We suggest a better definition of certain concepts such as protracted and rounded shoulder because muscle impairment and treatment will be different in these cases. We also recommend standardizing the teaching method of posture and muscular chain assessment between instructors in different countries. Future studies are still needed to document if these qualitative observations can be verified with standardized quantitative tests to assess muscle flexibility.

## Conclusion

Muscular chain evaluation by physical therapists trained in GPR conforms with standards taught by GPR instructors. The inter-rater reliability of this kind of evaluation is higher among more experienced physical therapists. This kind of assessment may improve physical therapy practice by guiding the understanding of muscular impairments associated with posture alterations and in the selection of therapeutic exercises to improve posture. However, it may be necessary to clarify some posture concepts and to standardize the assessment of some posture indices to increase inter-rater reliability.

## Competing interests

The authors report no conflict of interest.

## Authors’ contributions

CF and DF designed the study. CF was responsible for data collection. MH and CF were responsible for data analysis. CF, DF and MH contributed to data interpretation. CF and DF drafted the manuscript. CT critically revised the manuscript. All authors revised and approved the final version of the manuscript.

## Author disclosures

Funding : This project was supported by the Ordre Professionnel de la Physiothérapie du Québec and the Réseau Provincial de Recherche en Adaptation Réadaptation du Québec (OPPQ-REPAR). C. Fortin was supported by a Ph.D. scholarship from the Fonds de la recherche en Santé du Québec (FRSQ), MENTOR, a strategic CIHR training program/REPAR and research Centre of Sainte-Justine University Hospital Center. Dr Ehrmann Feldman is currently funded by the FRSQ.

## Pre-publication history

The pre-publication history for this paper can be accessed here:

http://www.biomedcentral.com/1471-2474/13/80/prepub
